# The kinetochore protein, *CENPF*, is mutated in human ciliopathy and microcephaly phenotypes

**DOI:** 10.1136/jmedgenet-2014-102691

**Published:** 2015-01-06

**Authors:** Aoife M Waters, Rowan Asfahani, Paula Carroll, Louise Bicknell, Francesco Lescai, Alison Bright, Estelle Chanudet, Anthony Brooks, Sonja Christou-Savina, Guled Osman, Patrick Walsh, Chiara Bacchelli, Ariane Chapgier, Bertrand Vernay, David M Bader, Charu Deshpande, Mary O’ Sullivan, Louise Ocaka, Horia Stanescu, Helen S Stewart, Friedhelm Hildebrandt, Edgar Otto, Colin A Johnson, Katarzyna Szymanska, Nicholas Katsanis, Erica Davis, Robert Kleta, Mike Hubank, Stephen Doxsey, Andrew Jackson, Elia Stupka, Mark Winey, Philip L Beales

**Affiliations:** 1 Institute of Child Health, University College London,London, UK; 2 Department of Nephrology, Great Ormond Street Hospital NHS Foundation Trust, London, UK; 3 Institute of Genetics & Molecular Medicine, Edinburgh, UK; 4 University of Massachusetts, Boston, USA; 5 Department of Cell and Developmental Biology, Vanderbilt University, USA; 6 Department of Clinical Genetics, Evelina Children's Hospital, London, UK; 7 Centre for Nephrology, Royal Free Hospital, University College London, London, UK; 8 Department of Clinical Genetics, Oxford Radcliffe Hospitals NHS Trust, Churchill Hospital, Oxford, UK; 9 Department of Medicine, Boston Children's Hospital and Harvard Medical School, Boston, USA; 10 Department of Pediatrics, University of Michigan, Ann Arbor, Michigan, USA; 11 Department of Pediatrics, Leeds Institute of Biomedical and Clinical Sciences, Leeds, UK; 12 Center for Human Disease Modeling, Department of Cell Biology, Duke University Medical Center; 13 MRC Human Genetics, University of Edinburgh, Edinburgh, UK; 14 Molecular, Ceullular, and Developmental Biology, University of Colorado at Boulder, Boulder, CO 80309, USA

**Keywords:** Clinical genetics, Molecular genetics, CENPF, Ciliopathy, Microcephaly

## Abstract

**Background:**

Mutations in microtubule-regulating genes are associated with disorders of neuronal migration and microcephaly. Regulation of centriole length has been shown to underlie the pathogenesis of certain ciliopathy phenotypes. Using a next-generation sequencing approach, we identified mutations in a novel centriolar disease gene in a kindred with an embryonic lethal ciliopathy phenotype and in a patient with primary microcephaly.

**Methods and results:**

Whole exome sequencing data from a non-consanguineous Caucasian kindred exhibiting mid-gestation lethality and ciliopathic malformations revealed two novel non-synonymous variants in *CENPF*, a microtubule-regulating gene. All four affected fetuses showed segregation for two mutated alleles [IVS5-2A>C, predicted to abolish the consensus splice-acceptor site from exon 6; c.1744G>T, p.E582X]. In a second unrelated patient exhibiting microcephaly, we identified two *CENPF* mutations [c.1744G>T, p.E582X; c.8692 C>T, p.R2898X] by whole exome sequencing. We found that CENP-F colocalised with Ninein at the subdistal appendages of the mother centriole in mouse inner medullary collecting duct cells. Intraflagellar transport protein-88 (IFT-88) colocalised with CENP-F along the ciliary axonemes of renal epithelial cells in age-matched control human fetuses but did not in truncated cilia of mutant *CENPF* kidneys. Pairwise co-immunoprecipitation assays of mitotic and serum-starved HEKT293 cells confirmed that IFT88 precipitates with endogenous CENP-F.

**Conclusions:**

Our data identify *CENPF* as a new centriolar disease gene implicated in severe human ciliopathy and microcephaly related phenotypes. CENP-F has a novel putative function in ciliogenesis and cortical neurogenesis.

## Introduction

Centrioles are microtubule (MT)-derived structures that play an essential role in centrosome and cilia formation.^1^ Mutations in centrosomal and MT-regulating genes have been described in cancer, disorders of neuronal migration^2^ ^3^ and microcephaly (MCPH) including Majewski osteodysplastic primordial dwarfism type II.^4^ ^5^ Disrupted processes include abnormalities of centriole duplication, centrosome maturation and spindle pole formation^6^ with defective asymmetric divisions of neuronal progenitors and failure of cortical neurogenesis.^7^ ^8^

Following mitosis, the distal appendages of the mother centriole become the transition fibres of the ciliary basal body.^9^ Transition fibres promote ciliogenesis by recruiting intraflagellar transport (IFT) proteins which traffic tubulin subunits and other proteins to the ciliary tip.^10^ Mutations in genes regulating centriole length have recently been described in ciliopathic disorders characterised by heterotaxia, retinal degeneration, skeletal dysplasia, renal disease and cerebral anomalies including microcephaly.^11–13^ These findings support emerging evidence that certain centriolar protein complexes have dual roles in spindle orientation and ciliogenesis.^8^ ^14^

In the current study, we identify *CENPF,* the MT-regulating gene, as a new centriolar disease gene implicated in severe human ciliopathy and MCPH-related phenotypes. Our data suggests a novel putative function for CENP-F in ciliogenesis as well as cortical neurogenesis.

### Methods

In order to determine the genetic basis of a novel congenital malformation disorder and MCPH, we employed a next-generation sequencing approach using whole exome sequencing combined with genome-wide linkage analysis.

### Research subjects

Approval for research involving human subjects was obtained from the Institute of Child Health research ethics board, University College London, and the Scottish multicentre research ethics committee.

### Linkage analysis

For genome-wide SNP mapping, the GeneChip Human Mapping 500 k Array from Affymetrix was used. Genotypes from DNA of the three affected and two unaffected children in the index kindred in addition to the parents were generated. Genotypes were examined with the use of a multipoint parametric linkage analysis, and haplotype reconstruction performed with GENEHUNTER V.2.1 through stepwise use of a sliding window with sets of 110 SNPs and the program, ALLEGRO, in order to identify regions of homozygosity as described using a disease allele frequency of 0.0001 and Caucasian marker allele frequencies.

### Exome capture

Targeted capture was performed on genomic DNA from one affected and one unaffected sibling of the index kindred with the EZ Exome Library (Roche Nimblegen, V.1) and sequenced on a single lane of a Solexa/Illumina Genome Analyser II. Reads were aligned to the human reference genome (GRC37 release, downloaded from the ENSEMBL database). Three different software programs were used for sequence evaluation: Maq, BWA and Novoalign. The coverage along the genome was calculated using BEDtools (GenomeCoverageBed function), without omitting zero values. Variant calling was undertaken using UnifiedGenotyper.^15^ The final output was then converted to variant call format. On average, we obtained about 43 million single short reads per lane with 91.8% of reads correctly mapped to the genome. The median sequencing depth per coding nucleotide was 23, with 90% of the targeted exons covered at least once. Variants from all samples were annotated and prioritised to identify pathogenic mutations as previously described.^16^ Variants annotated in dbSNP132 and the 1000 Genomes project or in our in-house databases with an allele frequency above 0.5% were removed. An autosomal recessive inheritance model was applied for gene identification in both kindreds, with known ciliopathy and MCPH genes manually analysed using the Integrative Genomics Viewer (http://www.broadinstitute.org/igv/). Candidate pathogenic variants were validated and assessed for familial segregation by Sanger sequencing.

### Sanger sequencing

Mutations were analysed by Sanger sequencing. *CENPF* primer pairs are described in online supplementary table S1.

### Immunofluorescence microscopy

NIH 3T3 fibroblasts, mouse inner medullary collecting duct (mIMCD3) and retinal pigmentary epithelial (RPE) cells were seeded onto glass coverslips and grown in Dulbecco’s modified eagle medium (DMEM) with 10% fetal bovine serum (FBS) and penicillin/streptomycin, until they reached 70% confluency, after which the medium was replaced with DMEM without serum overnight. Cells were stained with antibodies against CENP-F (1:200, ab 90, Abcam; sheep anti-CENP-F, 1:500, courtesy of Stephen Taylor, University of Manchester, rabbit anti-CENP-F,1:500, courtesy of Tim Yen, University of Pennsylvania), anti-IFT88 (1:800, 13967-1-AP Proteintech), anti-KIF3B (1:50, ab42494, Abcam), anti-α-acetylated-tubulin (1:800, T6793-clone 6-11B-1,Sigma-Aldrich), anti-γ-tubulin (1:500, T6557, Sigma-Aldrich), anti-GT335 (1:800, Novus Biologicals), anti-Ninein (1:200, ab 4447 Abcam), as previously described.^17^ Alexa-488, Alexa-594 and Alexa-647 conjugated secondary antibodies were obtained from Invitrogen. Confocal imaging was performed using a Zeiss LSM-710 system with an upright DM6000 compound microscope, and the images were processed with Zen software suite.

### Immunohistochemistry

Kidneys from 22-week-old control fetuses (phenotypically normal without karyotypical abnormality and normal kidney histology) and fetuses with *CENPF* mutations were fixed in 4% paraformaldehyde (PFA), dehydrated, embedded in paraffin and sectioned at 20 μm. Sections were stained with H&E. For immunofluorescent studies, microwave antigen retrieval and immunostaining were carried out as previously described.^18^ Confocal imaging was performed using a Zeiss LSM-710 system with an upright DM6000 compound microscope, and images were processed with Zen software suite. Z stacks were acquired at 0.5 μm intervals and converted to single planes by maximum projection with FiJi software.

### Electron microscopy

For immunogold labelling of RPE cells, the cells were serum-starved at 70% confluency for 3 days and then fixed in 0.25% glutaraldehyde +4% formaldehyde in 0.1 M cacodylate buffer, pH 7.4, and processed for embedment in LR White. Ultrathin sections (70 nm) were labelled with primary antibody, followed by secondary antibody conjugated to 12 nm gold particles.

### Co-immunoprecipitation studies

HEKT293, NIH 3T3 fibroblasts and RPE cells were plated onto tissue culture 100 mm dishes with 10 mL of DMEM medium containing 10% FBS and 1% penicillin/streptomycin. Cells were lysed after 48 h in a radio-immunoprecipitation assay buffer and were incubated with a monoclonal antibody to CENP-F (ab90, Abcam, 1:100) for 24 h at 4°C. After incubation, the lysates containing the Ag-Ab complexes were resolved by SDS-PAGE. Protein interactions were assessed by immunoblotting with affinity-purified polyclonal anti-IFT88 and anti-KIF3B antibodies (1:100). Enhanced chemiluminescence was used to detect specific proteins using secondary rabbit and mouse-conjugated horseradish peroxidase antibodies (dilution 1:2000).

### Gel filtration studies

Gel filtration was performed as previously described.^19^

### Zebrafish studies

Wild-type zebrafish, from AB×Tupfel long fin and transgenic cardiac myosin light chain (*cmlc2)*; *GFP*—zebrafish were staged and housed as previously described.^20^ Groups of 25–50 stage-matched embryos were collected at 8-somite and 18-somite stages, 24, 48, 72 and 120 h postfertilisation (hpf). For *cenpf* knockdown, antisense morpholino oligonucleotides (MO) (GeneTools) were designed against the 25 bps upstream of transcript start codon of *cenpf* and against the splice junction of the intron 3–exon 4 boundary (see online supplementary table S2). For controls, a standard control MO (5′-CCT CTT ACC TCA GTT ACA ATT TAT A 3′) was injected into wild-type embryos. Specificity of splice MO was confirmed by RT-PCR (see online supplementary figure S1). RNA was extracted from 25 morphants and 25 controls at 48 hpf using the TRIzol (Invitrogen) method. First-strand cDNA was synthesised using random hexamers (Sigma-Aldrich) and Omniscript transcriptase (QIAGEN), according to the manufacturer's instructions. Fertilised eggs were injected with MO (2 ng/embryo) at the 1-cell to 2-cell stage and allowed to develop at 28.5°C to the desired stages. For rescue experiments, full-length human CENPF plasmids were linearised with Not1 and mRNA synthesised using Ambion mMessage mMachine SP6 kit. Wild-type mRNA (75 pg) was injected into the cytosol of one-cell-stage embryos with *cenpf* MO. For whole-mount in situ hybridisation (WISH) studies, groups of 25–50 stage-matched embryos were collected at 18-somite stages and were fixed in 4% PFA/phosphate buffered saline overnight at 4°C. WISH for *southpaw* mRNA expression was undertaken with standard protocols.

### Statistical analysis

Statistical analyses were performed in GraphPad Prism V.5 (GraphPad Prism Software, USA). Numbers were reported as median values and comparison was made using the two-sample Wilcoxon rank sum test for non-parametric data. Where numbers are reported as mean values, comparison was made using the Student t test for parametric data. p<0.05 was considered statistically significant.

## Results

### Mutations in human *CENPF* cause a novel congenital malformation syndrome and MCPH

**Figure 1:**
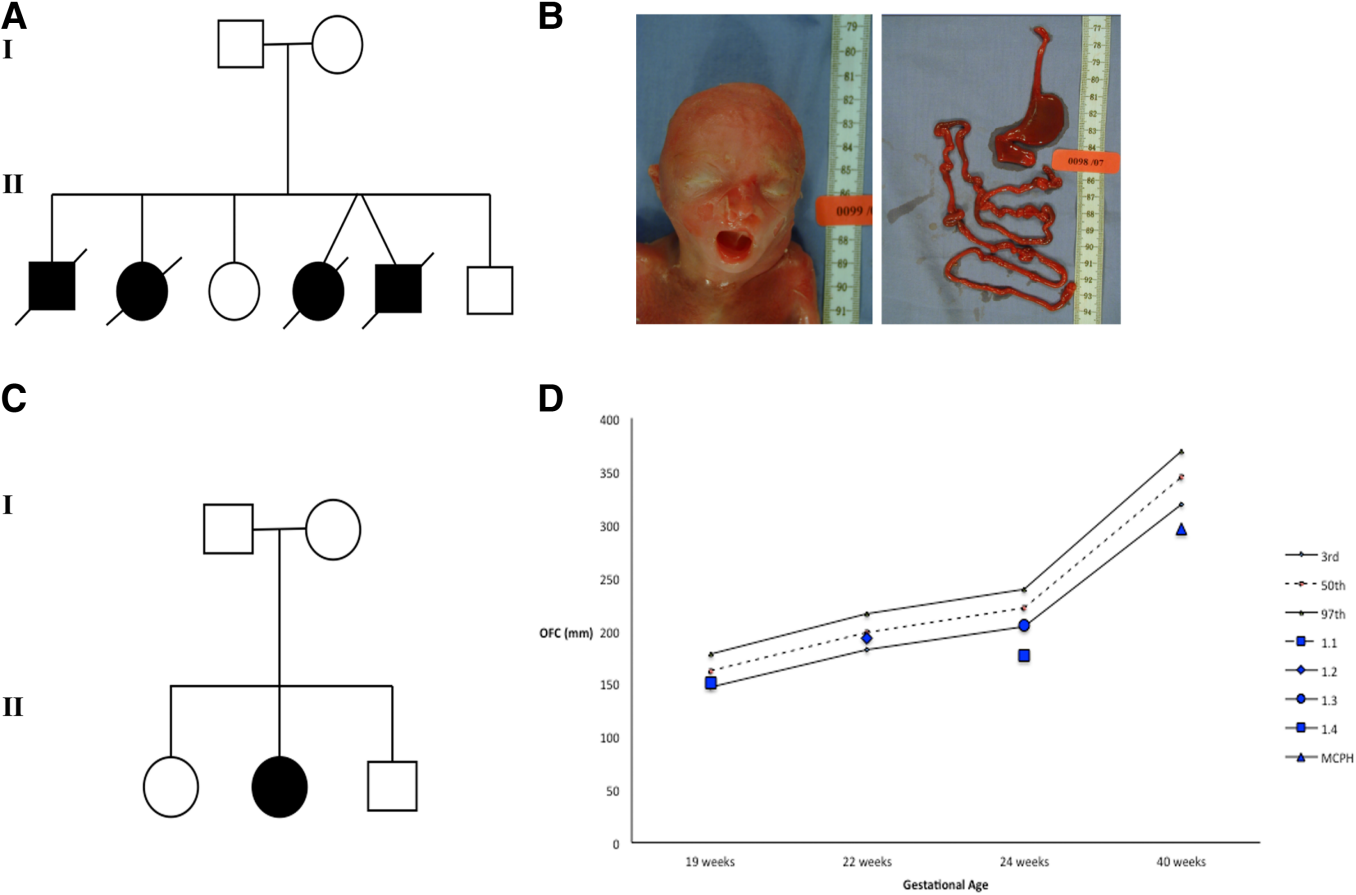
Pedigrees and clinical features of families with *CENPF* mutations. (A) The pedigree shows Family 1 with novel ciliopathy disorder consisting of non-consanguineous unaffected parents with six offspring, of which four were affected and died in utero, and two were unaffected and are healthy. (B) Gross morphological features of an affected fetus with dysmorphic craniofacial features such as a high nasal bridge, short columella, micrognathia, wide mouth and low-set ears. Examination of a dissected gastrointestinal tract from the same affected fetus revealed complete duodenal atresia. (C) The pedigree of Family 2 with a single affected case of microcephaly (MCPH) and unaffected parents and two unaffected siblings. (D) Chart demonstrating the occipital head circumference (OFC) data for the affected fetuses of kindred 1 (1.1, 1.3 and 1.4, at or below 3rd percentile) at gestational age at the time of autopsy in addition to the OFC at birth for the patient with MCPH of Family 2 which is below the 3rd percentile at 40 weeks gestation.

We identified a non-consanguineous Caucasian kindred with four affected fetuses exhibiting mid-gestation lethality and dysmorphic craniofacial features (figure 1A, B; table 1). Autopsy findings revealed ciliopathy features, such as cerebellar vermis hypoplasia, corpus callosum agenesis, cleft palate, duodenal atresia and bilateral renal hypoplasia.

**Table 1:** Clinical characteristics of index kindred with novel ciliopathy phenotype

Pedigree ID	Cerebral	Craniofacial	Gastrointestinal	Genitourinary
I.1* XY TOP 21 weeks	Hydrocephalus Cerebellar hypoplasia Agenesis of corpus callosum	Cleft palate Micrognathia Rounded head Low-set ears	Duodenal atresia	Bilateral renal hypoplasia
I.2* XX IUD 17 weeks	Hydrocephalus	Prominent nose High nasal bridge Short columella Wide mouth	Duodenal atresia	Bilateral renal hypoplasia
I.3 XX	Normal	Normal	Normal	Normal
I.4* XX Twin1 IUD 22 weeks	Hydrocephalus Agenesis of corpus callosum	Cleft palate	Duodenal atresia Malrotation Accessory spleens	Bilateral renal hypoplasia
I.5* XY Twin 2 IUD 22 weeks	Hydrocephalus Agenesis of corpus callosum	Microcrania Hypertelorism Broad nasal root Low-set ears	Duodenal atresia Multiple SI atresia Malrotation	Bilateral renal hypoplasia
I.6 XY	Normal	Normal	Normal	Normal

*Affected.

SI, small intestine.

**Figure 2:**
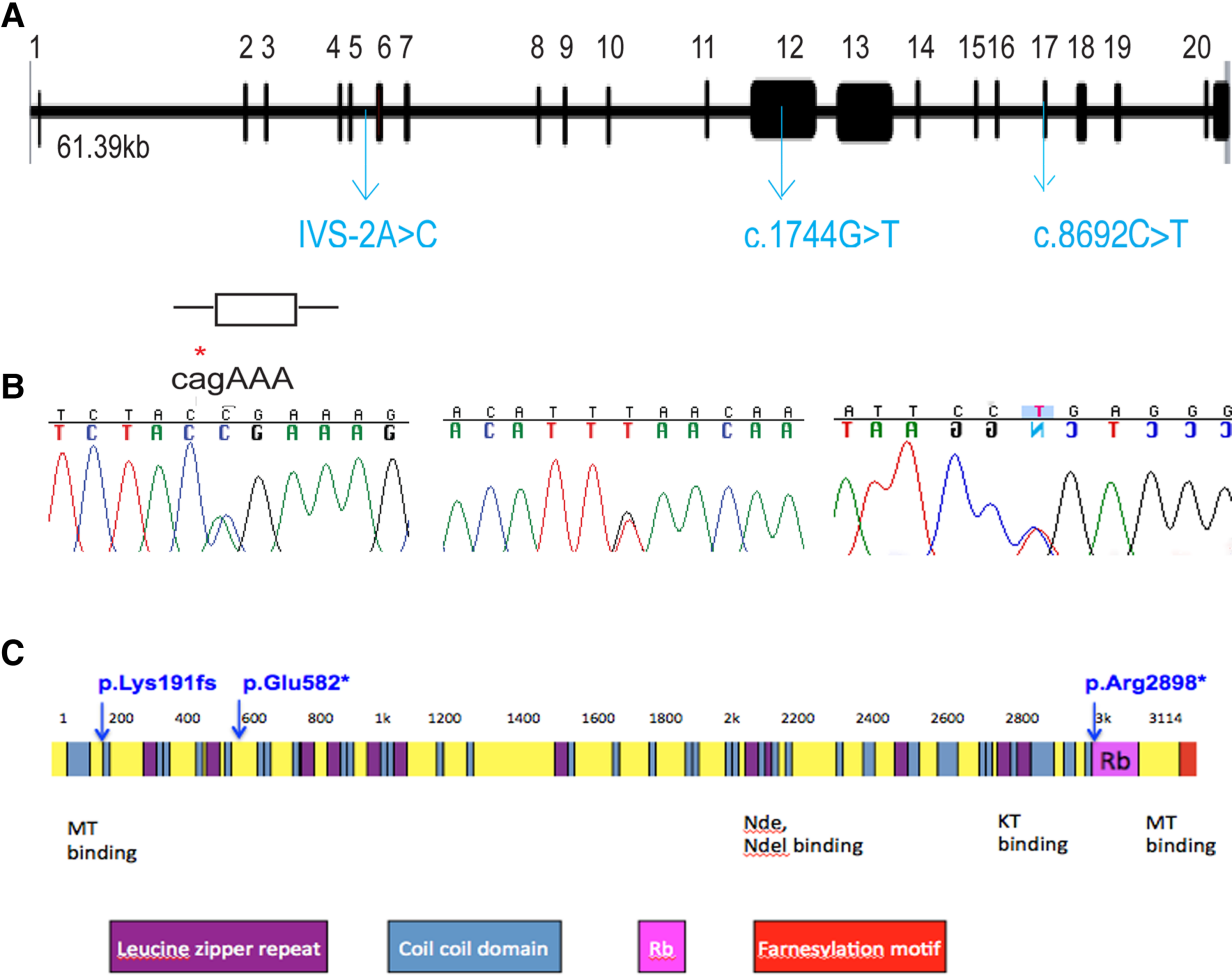
(A and B) *CENPF* genomic organisation, depicting locations of the identified heterozygous essential splice site non-synonymous mutation, IVS5-2A>C, and the heterozygous non-synonymous non-sense mutations, c.1744G>T and c.8692C>T (blue). For details on segregation, see also online supplementary figures S3 and S4. (C) *CENPF* encodes a protein of 350 kDa, consisting of 3114 amino acid residues. CENP-F protein consists of mainly coiled coil domains (blue), several leucine heptad repeats (purple), microtubule (MT)-binding domains at both the N and C termini in addition to Nudel (Nde) binding, kinetochore (KT)-binding and Nup133-binding domains. The kinetochore localisation domain and a bipartite nuclear localisation sequence reside in the C-terminal region.

A search of several validated dysmorphology databases including the London Dysmorphology Database failed to show phenotypical identity with any known syndrome. Genome-wide SNP analysis using high-density SNP arrays (Affymetrix 500 k Marshfield V.2) was undertaken on all except one family member (CIL 1.1). Linkage analysis using GENEHUNTER V.2 lr5 (multipoint) revealed 10 regions with a maximum positive HLOD of 1.32. Linked intervals were identified on chromosome 1 (2 intervals), 2, 6, 7, 8, 13, 19 and 20. One of the intervals on chromosome 1 and the interval on Chromosome 19 were the largest and contained the most homozygous markers covering a total of 839 genes (see online supplementary figure S2).^21^ Using Identity by Descent Finder, significant regions of homozygosity were not present, consistent with declared non-consanguinity. Given that the linked regions were large, spanning up to 15 Mb on 19p13.3, whole exome capture and consecutive massive parallel deep sequencing of one affected and one unaffected offspring was employed as a strategy to identify the underlying genetic aetiology of this novel phenotype. Variants were prioritised for analysis on the basis of novel coding non-synonymous SNPs, splice variants, truncating variants and InDels (see online supplementary table S3). A further variant filtering strategy based on an autosomal recessive mode of inheritance, as suggested by the pedigree, identified two novel homozygous and 40 novel compound heterozygous mutations in 20 genes that were unique to the affected offspring. Only one of the 22 candidate genes was present in a linked interval located on chromosome 1. Two novel non-synonymous variants in the *CENPF* gene (NM_016343.3), involving a heterozygous IVS5-2A>C nucleotide change, which was predicted to abolish the consensus splice-acceptor site from exon 6, and a second heterozygous c.1744G>T nucleotide change in exon 12 were identified (figure 2A, B). Sanger sequencing of both variants confirmed segregation with affected offspring and revealed that each parent carried a single variant (see online supplementary figure S3A). The variant was detected neither in the 200 ethnically-matched control alleles nor in the 200 control in-house exomes. The protein-truncating non sense variant, c.1744G>T, p.E582*, was predicted to be disease-causing in the Mutation Taster programme (score 6.0). The expected damaging effect provides strong support for its likely pathogenic effect. Furthermore, the mutated amino acid sequence is conserved among vertebrates (see online supplementary figure S3B). The canonical splice-site variant (IVS5-2A>C) disrupts the exon 6 acceptor splice site probably ablating exon 6 and leading to a 97 amino acid in-frame deletion affecting the MT-binding domain at the N-terminus of the protein. Another possible consequence of this mutation is that a cryptic splice site might be recruited downstream with resulting frameshift and the introduction of a premature stop codon (p.Lys191fs*). Non-sense-mediated decay would probably lead to loss of function. As all four affected fetuses died *in utero*, there was no RNA available to determine the effect of the canonical splice-site variant (IVS5-2A>C). Human *CENPF* consists of 20 coding exons that encode at least two protein-coding transcripts (see online supplementary table S4). Neither mutation is present in the second protein-coding transcript which overlaps its sequence with amino acids 2610–2774. The longest coding transcript of *CENPF* encodes a 350 kDa protein consisting of 3114 amino acid residues and mainly coiled coil domains. Other domains include MT binding domains at both the N and C termini, in addition to Nudel (Nde) binding, activating transcription factor-4 (ATF4)-binding and Nup133-binding domains (figure 2C).

Nde-1, the protein product of the centrosomal gene, mutated in microcephaly, has previously been shown to interact with CENP-F.^22–26^ We, therefore, investigated, by whole exome sequencing, a large cohort of patients (n=1000) with microcephaly (MCPH) for *CENPF* mutations, where no deleterious or potentially pathogenic variants were identified in known MCPH genes (*CEP135*, *ASPM*, *WDR62*, *MCPH1*, *CEP152*, *STIL*, *CEP63* and *CENPJ*). We identified two *CENPF* mutated alleles [c.1744G>T, p.E582*, c.8692 C>T, p.R2898*] in a patient with MCPH associated with mild to moderate learning difficulties (occipital head circumference (OFC) at birth: 29.5 cm, below 0.4th centile; adult OFC 45.5 cm, below 0.4th centile, figures 1C, D and 2A, B; see online supplementary figure S4). The other body systems were unaffected and overall growth was normal. Of note, MCPH was evident by mid-gestation in affected fetuses of Family 1 (figure 1D). The protein-truncating non sense variant, c.8692 C>T, p.R2898* was predicted to be disease-causing in the Mutation Taster programme (score 6.0). The expected damaging effect provides strong support for its likely pathogenic effect. The mutation c.1744G>T, p.E582* was shared between the two kindreds. Western blot analysis of protein lysates from *CENPF* mutant MCPH fibroblasts revealed a much greater reduction in CENP-F protein levels compared with wild-type controls (see online supplementary figure S5). The presence of residual protein could be explained by incomplete nonsense-mediated decay. Truncation of the residual protein as a result of the p.R2898* mutation is possible but the estimated difference in molecular weight of 23.7 kDa (estimated by loss of 216 amino acids) was difficult to resolve by SDS-PAGE gel electrophoresis owing to the high molecular weight of CENP-F (see online supplementary figure S9).

Mutational screening did not identify mutations in *CENPF* in 12 consanguineous patients with Meckel Grüber syndrome (MKS) who had compatible clinical features, potential autozygous regions and did not have a mutation in known *MKS* genes and in three patients with isolated nephronophthisis (NPHP) who showed homozygosity by descent at the *CENPF* locus out of a cohort of 150 families with NPHP or Joubert syndrome (JBTS). We also conducted mutational screening of 96 unrelated patients with Bardet Biedl syndrome (BBS) who were not preselected based on known BBS mutations. We did not detect recessive *CENPF* mutations in any of the MKS, NPHP, JBTS or BBS families, and because of the small sample size, we were unable to conclude that the heterozygous mutations in *CENPF* detected in BBS pedigrees act as phenotypical modifiers as has been described for other ciliopathy disease genes (see online supplementary table S5).

### CENP-F is localised to the subdistal appendages of the mother centriole

**Figure 3:**
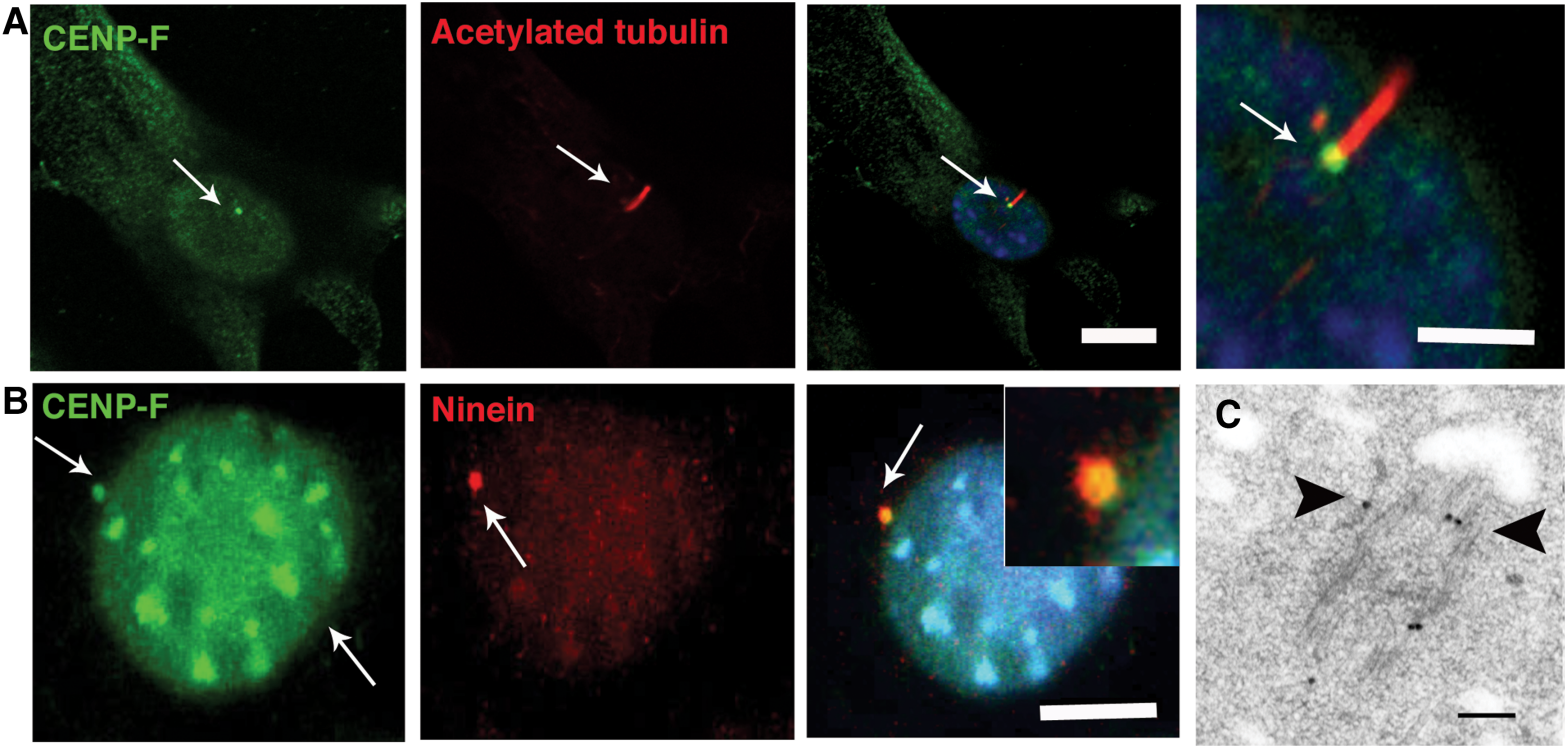
CENP-F is expressed at basal bodies of ciliated cells. (A) Shown are representative micrographs of cilia following dual immunofluorescence labelling of ciliated NIH 3T3 fibroblasts with anti-CENPF and anti-α-acetylated tubulin antibodies which demarcates cilia. CENP-F is localised to the basal bodies (arrows) of ciliated NIH 3T3 fibroblasts. Scale bar, 10 μm. (B) CENP-F colocalises with Ninein (arrows) at the subdistal appendages of the mother centriole of ciliated IMCD3 cells. Scale bar 5 μm. (C) Ultrastructural localisation of CENP-F in serum-starved retinal pigmentary epithelial cells. Black arrows point to immunogold particles along the microtubules and subdistal appendages of the mother centriole. Scale bar 100 nm.

The findings of mid-gestation fetal lethality together with abnormal craniofacial, cerebellar, palate, foregut and renal development, and the known role of Hedgehog signalling in organogenesis,^27^ suggested that these features may represent a ciliopathy disorder. Immunofluorescence microscopy of ciliated NIH 3T3 fibroblasts revealed a basal body localisation for CENP-F (figure 3A). Furthermore, CENP-F colocalised with Ninein at the subdistal appendages of the mother centriole of mIMCD3 cells (figure 3B). Transmission electron microscopy following immunogold labelling of CENP-F in serum-starved human RPE cells confirmed a centriolar localisation for CENP-F at the subdistal appendages and the distal end of the mother centriole (figure 3C).

### Zebrafish *cenpf* knockdown results in ciliopathy phenotypes

**Figure 4R2:**
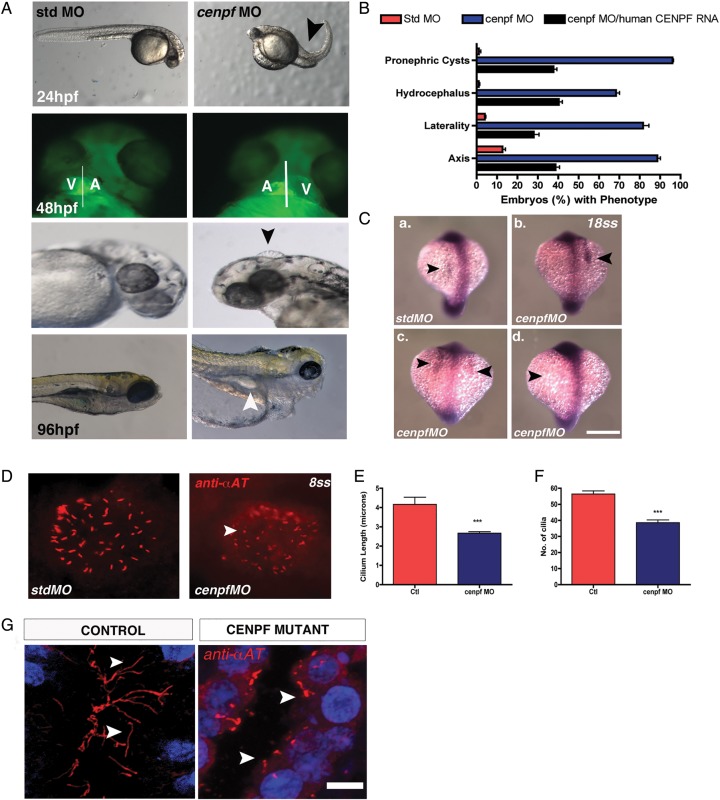
(A) Zebrafish *cenpf* morphants display increased body axis curvature at 24 h postfertilisation (hpf) compared with control embryos (black arrow). *Cenpf* knockdown in *cardiac myosin light chain (cmlc2)-gfp* transgenic zebrafish causes laterality heart defects at 48 hpf. Hydrocephalus (arrow) is evident at 72 hpf in *cenpf* morphants compared with control embryos. At 96 hpf, pronephric cysts (arrow) are evident in *cenpf* morphants compared with control embryos. (B) Quantitative graph showing increased occurrence of axis curvature defects, laterality malformations, hydrocephalus and pronephric cysts in *cenpf* morphants (blue bars) compared with control embryos (red bars) and compared with *cenpf* morphants injected with human *CENPF* RNA (black bars). Bars represent an average of three experiments. Error bars denote SE of the mean (SEM). [Std-MO (n=266) % ventral axis curvature at 24 hpf vs *cenpf-*MO (n=173) 12.7±1.5 vs 88.7±1.4, *p<0.001; *cenpf-*MO (n=173) vs *cenpf-*MO with human *CENPF* RNA (n=256) 88.7±1.4 vs 38.7±2.0, *p<0.001; Std-MO (n=223) % laterality defects at 48 hpf vs *cenpf-*MO (n=152) 4.0±0.6 vs 81.7±2.8, *p<0.001; *cenpf-*MO (n=152) vs *cenpf-*MO with human *CENPF* RNA (n=229) 81.7±2.8 vs 28±2.6, ***p<0.01; Std-MO (n=204) % hydrocephalus at 72 hpf vs *cenpf-*MO (n=93) 1±0.6 vs 68.3±1.7, *p<0.001; *cenpf-*MO (n=93) vs *cenpf-*MO with human *CENPF* RNA (n=197) 68.3±1.7 vs 40.3±1.7, *p<0.001; Std-MO (n=158) % pronephric cysts at 120 hpf vs *cenpf-*MO (n=76) 1.2±0.9 vs 96±0.6, ****p<0.0001; *cenpf-*MO (n=76) vs *cenpf-*MO with human *CENPF* RNA (n=122) 96±0.6 vs 37.3±1.9, *p<0.001]. (C) Representative images of *southpaw* mRNA expression in the lateral plate mesoderm at 18-somites (ss) of control (a) and *cenpf* morphant embryos (b-d). (a) left-sided expression in control embryos (arrow, top left panel). (b) right-sided expression (arrow), (c) bilateral expression and (d) absent expression in stage-matched *cenpf* morphant embryos (arrows). Scale bar 50 μm. (D) Representative micrographs following immunofluorescent labelling of Kupffer's vesicle (KV) cilia with anti-α-acetylated tubulin antibody at 8 ss. Short KV cilia are noted in *cenpf* morphants (white arrows) (E) Quantitative graph showing a quantitative difference in KV cilia length (µm) in *cenpf* morphants (n=136 cilia; n=5 embryos) vs controls (SD MO) (n=228 cilia; n=4 embryos); 4.2±0.4 vs 2.6±0.1 **p<0.0001). (F) Quantitative graph showing that KV cilia number were significantly less in *cenpf* morphants (n=5 embryos) vs controls (std MO) (n=5 embryos); 56.4±1.9 vs 38.6±1.7 **p<0.001). (G) Long cilia are observed in the lumina of collecting ducts of control fetuses (white arrow) while short cilia are evident on renal epithelial cells of *CENPF* mutant fetal kidneys (white arrow). Sections are counterstained with 4′,6-diamidino-2-phenylindole. Scale bar 10 μm. MO, morpholino oligonucleotides.

To understand the functional relevance of CENP-F in relation to its localisation at the basal body, we performed knockdown experiments using both translation-blocking and splice-blocking antisense MO against zebrafish *cenpf* which shares 60% homology with its human orthologue (see online supplementary table S2). Compared with controls, a significantly reduced number of *cenpf* morphants survived (p<0.008, online supplementary figure S1). Morphological analysis of surviving zebrafish embryos at 24 hpf, revealed axis curvature defects and at 48 hpf, *cenpf* morphants exhibited abnormal heart looping compared with controls (figure 4A). At 72 hpf, hydrocephalus was observed in *cenpf* morphants, and at 120 hpf, all surviving *cenpf* morphants exhibited pronephric cysts (figure 4A). Coinjection of *cenpf* MO with human wild-type *CENPF* RNA rescued significantly the axis curvature defects (p<0.001) and pronephric cysts in *cenpf* morphants (p<0.001; figure 4B, online supplementary figure S6 and table S5). The occurrence of these morphological findings support a role for CENP-F in zebrafish ciliary function.^28^

Cilia-driven fluid flow within zebrafish Kupffer's vesicle (KV), or across the mouse ventral node, has been shown to underlie a conserved symmetry-breaking event that establishes a left–right (LR) pattern.^29^ To corroborate our findings of early lethality and abnormal heart looping in embryos surviving to 48 hpf, we next determined whether LR patterning defects are evident in *cenpf* morphants. At mid-somite stages, normal LR patterning can be defined by *southpaw* mRNA expression in the left lateral plate mesoderm.^29^ In *cenpf* morphants, right-sided (top right panel), bilateral (bottom left panel) and absent (bottom right panel) expression of *southpaw* mRNA was observed compared with left-sided expression in control embryos (figure 4C and also see online supplementary figure S7). To determine whether the laterality defects are caused by defects in KV cilia, we next analysed cilia formation in 8-somite-stage (ss) control and *cenpf* morphants. Following labelling of KV ciliary axonemes with anti-α-acetylated tubulin antibody in stage-matched embryos at 8-somites (figure 4D), analysis of *cenpf* morphants revealed that the length of KV cilia were shorter compared with controls (p<0.0001; figure 4E) and the number of KV cilia was significantly less in *cenpf* morphants compared with controls (p<0.001; figure 4F).

Following our observations that *cenpf* morphants show defective KV ciliogenesis, we next determined whether defective ciliogenesis was a feature of mutant *CENPF* renal epithelia. Immunofluorescence labelling of ciliary axonemes with anti-α-acetylated tubulin antibody in kidney sections of autopsy tissue from age-matched control fetuses were longer compared with those in renal epithelia of fetuses carrying the identified *CENPF* mutations (figure 4G). While renal epithelial cilia were noted to be present on some but not all cells, morphologically, the cilia that were present were stumpy with a terminally distended appearance (figure 4G).

### CENP-F colocalises with IFT88 along the ciliary axoneme and precipitates with endogenous IFT88 and other IFT-B components

**Figure 5:**
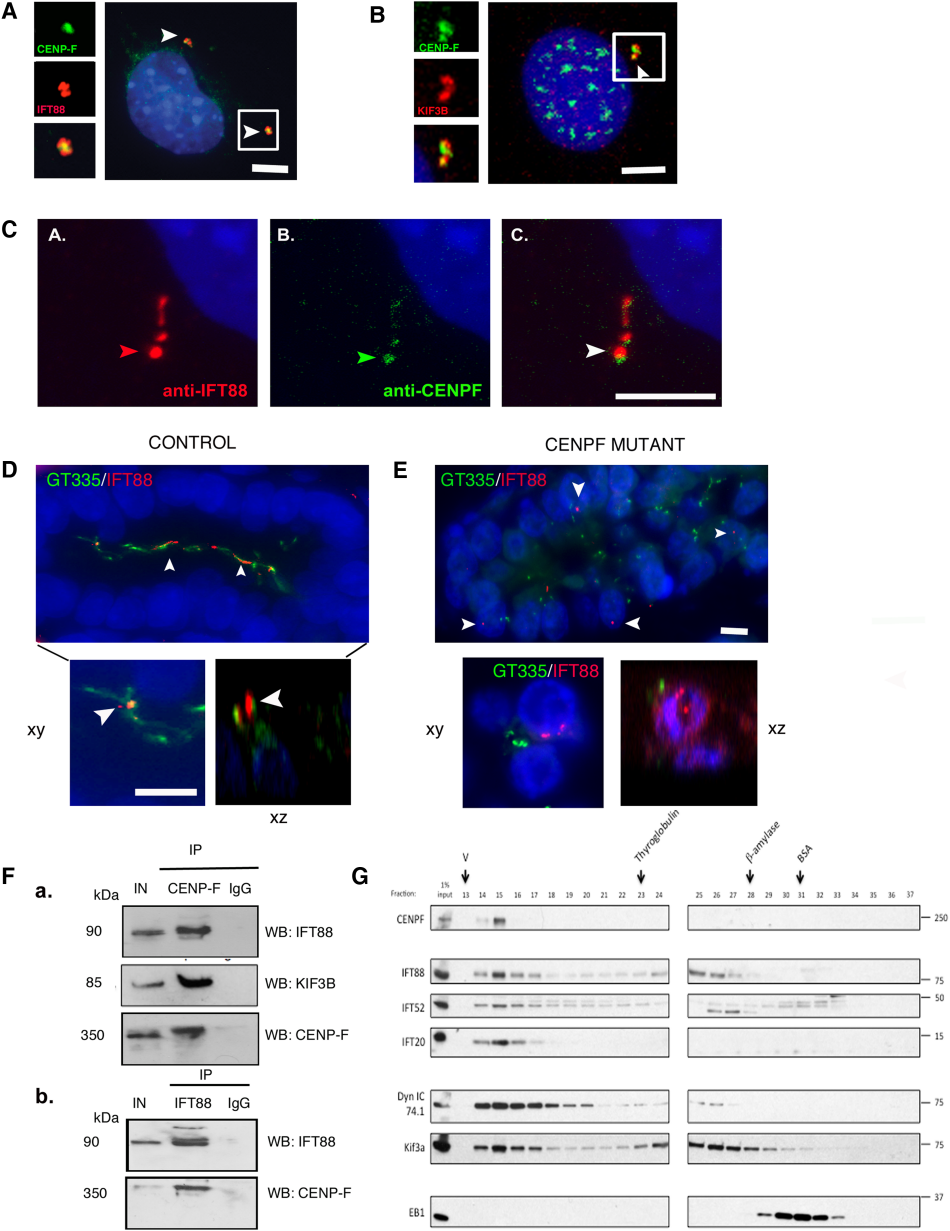
(A) Colocalisation of CENP-F at the centrosome with intraflagellar transport-88 (IFT88) (B) Representative micrographs of asynchronous 3T3 fibroblasts following dual immunofluorescent labelling of 3T3 cells with KIF3B and C-terminus CENP-F antibody. CENP-F localises to the centrioles with KIF3B (arrows). Scale bar 5 μm. Inset: high-power view of CENP-F localisation between two KIF3B foci. (C) Co-localisation of CENP-F along ciliary axonemes labelled with IFT88 antibody (D) IFT88 localises to long cilia within the lumina of renal collecting ducts of 22-week-old control human fetuses (arrows). Inset shows colocalisation of IFT88 with GT335-positive ciliary axonemes in xy and xz plane of confocal projection. Scale bar xy, 15 µm. (E) IFT88 does not localise with GT335-positive ciliary axonemes, if present, of *CENPF* mutant fetal kidneys (arrows). GT335-positive cilia are shorter than cilia in control kidneys. Scale bar 10 μm. Inset shows that IFT88 and GT335 do not colocalise in the xy and xz plane of confocal projection. Scale bar 10 μm. (F) Representative images of co-immunoprecipitation experiments carried out on protein lysates from mitotic HEKT293 cells containing endogenous CENP-F. Immunoblots show that IFT88, KIF3B and CENP-F co-immunoprecipitate with endogenous CENP-F, while an immunoglobulin G (IgG) isotype control does not co-immunoprecipitate with CENP-F. IN=input; 10% of total input is indicated. (b) Reciprocal co-immunoprecipitation experiments carried out on protein lysates from serum-starved HEKT293 cells containing endogenous IFT88. Immunoblots show that CENP-F co-immunoprecipitates with endogenous IFT88, while an IgG isotype control does not co-immunoprecipitate with IFT88. IN=input; 10% of total input is indicated. (G) Asynchronous HeLa cell lysate was fractionated over a superose-6 gel filtration column. Eluted fractions were probed with antibodies against CENP-F, IFT complex B members: IFT88, IFT52 and IFT20, and motors: cytoplasmic dynein 1 intermediate chain (Dyn IC 74.1) and Kif3a. CENP-F co-eluted with the IFT proteins and motors, suggesting that it exists as a complex with these proteins. Arrows indicate peak elution fractions for calibration proteins: thyroglobulin (669 KDa; fraction 23), β-amylase (200 KDa, fraction 28) and bovine serum albumin (BSA) (67 KDa, fraction 31). V, void volume.

Previous work has demonstrated that during mitosis, CENP-F localises to the spindle poles in a process that relies on cytoplasmic dynein-1.^22–24^ ^30^ Similar findings for IFT88 have recently been reported,^19^ suggesting that ciliary proteins may have specific roles in mitosis.^19^ ^31^ Consequently, we tested the hypothesis that CENP-F might interact with IFT88. To test this, we showed that CENP-F colocalised with IFT88 and KIF3B at the centrosome of asynchronous NIH 3T3 fibroblasts (figure 5A, B) and along the ciliary axonemes of ATDC5 cells (chondrocytes) (figure 5C) Furthermore, in human autopsy samples, IFT88 colocalised with ciliary axonemes of renal epithelial cells of control fetuses (figure 5D). However, IFT88 did not localise to ciliary axonemes and centrioles in mutant *CENPF* kidneys (figure 5E). These data prompted us to test a possible functional relationship between CENP-F and IFT88. Pairwise co-immunoprecipitation assays of mitotic and serum-starved HEKT293 cells confirmed that IFT88 precipitates with endogenous CENP-F (figure 5F). In unsynchronised HeLa cells, endogenous CENP-F co-fractionated with other IFT-B components such as IFT52 and IFT20 in addition to motors, such as cytoplasmic dynein-1 and Kif3a (figure 5G). Therefore, this data suggest that CENP-F interacts with proteins involved in cilia formation and function.

Together with our findings that *CENPF* mutations result in a microcephalic phenotype and recent evidence for a role for IFT88 and Pericentrin in mitotic spindle orientation,^8^ ^19^ we hypothesised that CENP-F may also interact with proteins implicated in the cortical polarity pathway which could account for defective cortical neurogenesis. This hypothesis was supported by co-immunoprecipitation assays suggesting an interaction between CENP-F and the NuMA/p150 Glued dynactin/Par 3 protein complex, proteins implicated in asymmetric cell division^32^ (see online supplementary figure S8).

## Discussion

### Mutations in *CENPF* link the kinetochore complex to human ciliopathy and MCPH phenotypes

Our discovery of a novel severe human ciliopathy-related phenotype attributed to mutations in a kinetochore protein, supports recent evidence for a dual role for ciliary proteins in spindle orientation and ciliogenesis.^19^ CENP-F was first characterised in cancer cell lines as a component of the outer kinetochore and as a binding partner of the retinoblastoma (Rb) protein.^33–35^ CENP -F is dynamically expressed throughout the cell cycle. It binds to the nuclear envelope at the transition between G2 and M phase of the cell cycle. In early prophase until anaphase onset, it is found at the kinetochore where it stabilises the attachment of MTs to the centromere. In early anaphase, CENP-F is found at the spindle mid-zone, while in late anaphase, it migrates with cytoplasmic dynein-1 and recruits the spindle checkpoint regulatory complex to the spindle poles.^22^ In early G0, it undergoes proteasome degradation.^36^ Depletion of CENP-F *in vitro* results in mitotic delay, with failure of kinetochore assembly and misalignment of chromosomes in a subset of mitotic cells.^37^ ^38^ Studies in murine embryonic stem cells and in avian myocyte lines suggest primary roles for this protein complex in differentiation, but the exact mechanism is unknown.^39^ Several studies have highlighted an additional role for CENP-F in the regulation of cell shape and vesicle transport.^23^ ^40–42^ Given the overlap of features in fetuses with *CENPF* mutations with human ciliopathy phenotypes, we hypothesised that CENP-F may play a role in cilia formation and function. The finding that CENP-F colocalises with IFT88 along the ciliary axoneme, and the existence of CENPF-IFT88 protein complexes is further supported by the co-migration of CENP-F with other IFT-B components. Furthermore, the absence of IFT88-stained ciliary axonemes and mislocalisation of IFT88 within renal tubular epithelia of *CENPF*-mutant kidneys proposes a putative role for CENP-F-dependent IFT88-ciliary targeting.

We attribute, for the first time, that mutations in the *CENPF* gene play a causal role in human congenital malformation syndromes. All four affected fetuses of the index kindred had compound heterozygous mutations in *CENPF*. Further supporting the pathogenicity of these mutations was the finding that one of these mutations also occurred in the context of MCPH in a second unrelated kindred. Phenotypical disparity is not a unique finding to mutations in the *CENPF* gene. Divergent phenotypes have been previously described in several disorders associated with mutations in genes encoding centrosomal proteins. For example, for mutations in the *CEP290* gene, 90 mutations have been reported exclusively in only one phenotype, while 14 others have segregated with two diseases, and eight have been associated with three or more phenotypes.^43^ In most cases, these phenotypes are partially overlapping, although few mutations were observed to lead to strongly divergent disorders, such as Leber's Congenital Amaurosis (LCA) and MKS. It is interesting that mutations causing JBTS tend to cluster in the second half of the *CEP290* gene, whereas mutations segregating with LCA, Senior Löken Syndrome and MKS are homogeneously distributed throughout the gene.^44^ ^45^ Of note, domains within the CEP290 protein share significant similarity with CENP-F, with several putative coiled-coil domains, a region with homology to structural maintenance of chromosomes segregation ATPases, a bipartite nuclear localisation signal and six RepA/Rep^+^ protein kinase inducible domain motifs and an ATF4-binding domain.^46^

In the current study, we sought to explain the divergent phenotypes by an analysis for potential modifier alleles in genes known to be associated with ciliopathy phenotypes, but did not find any variants which were predicted to change the amino acid sequence. A plausible explanation could be perhaps that the more severe phenotype in the embryonic lethal disorder was associated with a lack of a protein product necessary for ciliary function. Because residual CENP-F protein was observed in the fibroblasts of our MCPH patient, a dosage-dependent mechanism could be proposed in which complete loss of function of both *CENPF* alleles would lead to an embryonic lethal ciliopathy phenotype, whereas residual CENP-F activity would be sufficient for normal ciliary targeting of IFT88 and, perhaps, a less severe phenotype.

Genes involved in centrosome maturation and spindle-pole formation have been implicated in MCPH phenotypes.^6^ As CENP-F migrates with cytoplasmic dynein-1 and recruits the spindle checkpoint regulatory complex to the spindle poles in late anaphase,^22^ the findings of *CENPF* mutations in a MCPH phenotype is therefore not unexpected. MCPH phenotypes have been ascribed to defective asymmetric divisions of neuronal progenitors and failure of correct neural cell fate specification.^6^ ^8^ Our data supports a role for CENP-F in asymmetric cell divisions through its putative interactions with p150-dynactin, NuMA and Par3, proteins which have been implicated in this pathway.^32^ Future investigations using conditional *Cenpf*-deleted transgenic mice will be needed to dissect the role of CENP-F in cortical neurogenesis.

## Supplementary Material

Web supplement

Web figures
