# Erratum: The kinetochore protein, *CENPF*, is mutated in human ciliopathy and microcephaly phenotypes

**DOI:** 10.1136/jmedgenet-2014-102691corr1

**Published:** 2016-10-13

Waters AM, Asfahani R, Carroll P, Bicknell L, Lescai F, Bright A, Chanudet E, Brooks A, Christou-Savina S, Osman G, Walsh P, Bacchelli C, Chagpier A, Vernay B, Bader DM, Deshpande C, O'Sullivan M, Ocaka L, Stanescu H, Stewart HS, Hildebrandt F, Otto E, Johnson CA, Szymanska K, Katsanis N, Davis E, Kleta R, Hubank M, Doxsey S, Jackson A, Stupka E, Winey M, Beales PL. The kinetochore protein, *CENPF*, is mutated in human ciliopathy and microcephaly phenotypes. *J Med Genet* 2015;52:147–56. Published Online First: 6 January 2015 doi:10.1136/jmedgenet-2014-102691.

The following statement should have been included in the funding statement:

LSB was supported by Medical Research Scotland.

