# Supplementary material for: The kinetochore protein, CENPF, is mutated in human ciliopathy and microcephaly phenotypes
Source: J Med Genet. 2015 Jan 6;52(3):147–56. doi: 10.1136/jmedgenet-2014-102691 (PMC4345935; doi:10.1136/jmedgenet-2014-102691)
Supplement: Web supplement [file jmedgenet-2014-102691-s1.pdf]

SUPPLEMENTARY TABLES

TABLE S1. Oligonucleotide Primers used for *CENPF* Sequencing

Gene	Primer sequences (5' to 3')	Product size
CENPF Exon 2 F	GAAACTTGATTTTTAGGGGTGGT	324bp
CENPF Exon 2 R	AAATACCAGCACTTCTCTGTCAA	324bp
CENPF Exon 3 F	TGGCTTATTGCAGCTGTATCTC	417bp
CENPF Exon 3 R	ACGGTACAGAGACCGAATCA	417bp
CENPF Exon 4 F	CTCTGGGAATGTAAGGCATTG	387bp
CENPF Exon 4 R	GAATTTCTTTGAAAATATGCCACA	387bp
CENPF Exon 5 F	TGTGTTTTGATATTTGAGTAATTTGA	358bp

CENPF Exon 5 R	TGAGCCCAAAACCTTTTCTC	358bp
CENPF Exon 6 F	AACTTCTTGGGATTATGGCTTT	457bp
CENPF Exon 6 R	CGATGTGCCTAACAAAACACA	457bp
CENPF Exon 7 F	GAAAATCTGTTTCTCCTGCTTTC	546bp
CENPF Exon 7 R	CGGATCTGCCCAACTTAAAA	546bp
CENPF Exon 8 F	TTTTTCATGGCACAAATTAGGA	439bp
CENPF Exon 8 R	GCGCAAAAGTGAAGATGTGA	439bp
CENPF Exon 9 F	ACCTGGATTTGATGCCTGAG	448bp
CENPF Exon 9 R	GGAAACCTAGAGCCAGAATGG	448bp

CENPF Exon 10 F	GACGTCGCAAGGTCACATTA	443bp
CENPF Exon 10 R	GCAATCATATTCTGTCATGGGTTA	443bp
CENPF Exon 11 F	TTAATAGGCGTATGAACAATGAGAA	477bp
CENPF Exon 11 R	TTTCTCTAATATGTTAATGCCATCC	477bp
CENPF Exon 12A F	CCAAGTGATCCACTTTCTAGGAG	600bp
CENPF Exon 12A R	AACACGTTGTGAAGGTTTCTGA	600bp
CENPF Exon 12B F	AACTTGTCTGAAGACACAGCAAA	549bp
CENPF Exon 12B R	TTCATCTGTTTTTGAGACTCTAATGA	549bp
CENPF Exon 12C F	CTGCCATGCATCATTCCTTT	737bp

CENPF Exon 12C R	TCTTTTCCTGTGCTGCTTTG	737bp
CENPF Exon 12D F	CAGAGTTATCTGATCAGTACAAGCAA	672bp
CENPF Exon 12D R	TGCAAATTGCTGGTTTCAAG	672bp
CENPF Exon 12E F	CGCAGTTGGTGCAATTAGAA	413bp
CENPF Exon 12E R	CACCATGGAGAAGACCACTG	413bp
CENPF Exon 12F F	AAGAGGTAGGGAAACTACTAAATGAA	600bp
CENPF Exon 12F R	TCAGATTCTCCTCCTGCAGAC	600bp
CENPF Exon 12G F	TCTTGTGTGCCTGACAGCTC	662bp
CENPF Exon 12G R	TTGGTGTATTTTATTTCCTTGAACC	662bp

CENPF Exon 13A F	CCTGAATATTCTTAGCAAGGGAAA	592bp
CENPF Exon 13A R	CCCGCAGTTGAAGATTATGG	592bp
CENPF Exon 13B F	GAAACCCACAGGAGAGTGCT	456bp
CENPF Exon 13B R	TTCACGTGATGATTTATCTGCAT	456bp
CENPF Exon 13C F	CAGGAGGTACAACTAATGACCAAA	422bp
CENPF Exon 13C R	ATCCAGTGCCGTGGTTTTT	422bp
CENPF Exon 13D F	TGAGCATGAAGCCCTCTACC	453bp
CENPF Exon 13D R	TGCAGGCTTTCAGATTCCTT	453bp
CENPF Exon 13E F	GGCAGAGGTGAAGGAAAAGA	389bp

CENPF Exon 13E R	GCTCCTGGTTTTCTTCTGACA	389bp
CENPF Exon 13F F	ACACAGGAGGAAGTGCATCA	446bp
CENPF Exon 13F R	GGGCTCTCAGCTTTTCAATG	446bp
CENPF Exon 13G F	AAAACTGCAGTGGAGATGCTT	393bp
CENPF Exon 13G R	TTCGCTCTTGCTCTTTTTGTAA	393bp
CENPF Exon 13I F	AGCCCTGCATAATGACCAAG	478bp
CENPF Exon 13I R	TGGTTTCCTGCCTCATGACT	478bp
CENPF Exon 14 F	TGTTGTATCAGAGTGGTCGATCT	262bp
CENPF Exon 14 R	GGAACCAATAAGGAGAGTGTGC	262bp

CENPF Exon 15 F	TGTACAGATTTTATCTTG CCCATAA	413bp
CENPF Exon 15 R	CTGGGGAAAAGATCGTG AAG	413bp
CENPF Exon 16 F	ACTGCGCCCAGCTGTTTT	352bp
CENPF Exon 16 R	TGATGAATGACATCATTT TTGACT	352bp
CENPF Exon 17 F	CGTGAATGGTTTTGTGCA TC	432bp
CENPF Exon 17 R	GCACAAAATTCAGAAATT GGAA	432bp
CENPF Exon 18 F	CCCGAACAAGAGTTGTTT GAA	773bp
CENPF Exon 18 R	GGAAAACATATGCCTCAT CCA	773bp
CENPF Exon 19 F	TGACCACAGTGGCTAGG ACA	352bp

CENPF Exon 19 R	GTCCAATCCTCACCCAGGTA	352bp
CENPF Exon 20 F	GGGACGTCTGATGACTGGTT	454bp
CENPF Exon 20 R	TCCTGTAGGCACAGCCTTATC	454bp

TABLE S2: Morpholino sequences for zebrafish studies

Oligos	5'-3' sequence
ATG *cenpf* MO	TCCACTCTTCTACAGCCCAACTCAT
Splice *cenpf* MO	TGGAGTCTGAAAATGCAATATTTGA

TABLE S3: Prioritisation of variant analysis

	Foetus 1.2 (Affected)	Foetus 1.5 (Unaffected)
Total Reads	43, 376, 158	43, 379, 788
Mappable Reads	39, 220, 720	40, 429, 960
Mean Coverage (x)	29.95	24.27
Total variants called	42, 606	48, 254
Novel variants	10, 432	10, 970
Exonic + Disruptive Splice Site (Phred>50)	4, 112	4, 306
Nonsense, Disruptive Splice Site, Frameshift InDels & Missense	648	656
Homozygous	3	4
Compound Heterozygous	24 (48 variants)	17 (34 variants)
Unique to sample Homozygous	2	3

Unique to sample Compound Heterozygous	20 (40 variants)	12 (24 variants)
Linked regions	1	
Segregation analysis	1	

TABLE S4: Protein coding transcripts for *CENPF*

Name	Transcript ID	Length (bp)	Protein ID	Length (aa)
CENPF-001	ENST00000366955	10307	ENSP00000355922	3114
CENPF-201	ENST00000391896	525	ENSP00000375766	175

TABLE S5: *CENPF* variants identified in BBS patients

BBS patient	cDNA change	Amino acid change	NHLBI ESP (Aug 2012)	Other allele 1	Other allele 2
AR433-05	c.4582T>C	p.Cys1528Arg	C=5/T=13001	BBS10: S303fsX305	BBS10: G677V
AR198-04	c.5378G>A	p.Arg1793His	A=30/G=12976	BBS12: F372fsX373	BBS12: G540V
AR316-03	c.6926G>A	p.Arg2309His	A=19/G=12987	BBS12: E365fsX382	BBS12: X711Y
KK015-03	c.7904T>C	p.Leu2635Pro	0	BBS10: F275fsX281	

Table S5: Human *CENPF* RNA rescues ciliopathy phenotypes in zebrafish *cenpf* morphants

	Control	*cenpf* MO	*cenpf* MO h*CENPF* RNA
**Ciliopathy Phenotype**	**Total (n)* %****	**Total (n)* %****	**Total (n)* %****
**Ventral axis curvature**	(n=266) 12.7±1.5	(n=173) 88.7±1.4	(n=256) 61.3±2.1
**Pronephric cysts**	(n=158) 1.2±0.9	(n=76) 96±0.6	(n=122) 62.3±1.7

* Total number of embryos examined over 3 experiments

** Mean percentage of embryos ± standard error of the mean (S.E.M.)

SUPPLEMENTARY FIGURE LEGENDS

Figure S1

(A) RT-PCR of RNA from *cenpf* splice zebrafish morphants demonstrating specificity of splice morpholinos by aberrant splicing of *cenpf* mRNA in cenpf splice morphants compared to control embryos at 24 hpf. (B) Zebrafish *cenpf* morphants exhibit high mortality in first 24 hours. Quantification (%) of surviving zebrafish embryos injected with morpholino (MO) at 24hpf. Graphic representation of results showing the mean percentage of survival at 24hpf of standard-MO (control), 1ng *cenpf* MO and 2ngs *cenpf* MO. Bars represent an average of three experiments. Error bars denote standard error of the mean (S.E.M). [Std-MO (1ng, n=266) % surviving embryos at 24hpf vs. *cenpf*-MO (1ng, n=173) 85.7±1.2 vs. 46.7±0.9, * $p<0.008$; Std-MO vs. *cenpf*-MO (2ng, n=256) 85.7±1.2 vs. 36.3±2.3, ** $p<0.001$; *cenpf*-MO (1ng, n=173) vs. *cenpf*-MO (2ngs, n=256) 46.7±0.9 vs. 36.3±2.3, ns, $p<0.09$].

Figure S2: Linkage plot of multipoint linkage analysis in a kindred with novel ciliopathy phenotype. Ten chromosomal regions were identified with a positive LOD score. Plot derived from GENEHUNTER version 2 [21].

Figure S3: Segregation and Conservation of compound heterozygous mutations in *CENPF*.

(A) The heterozygous essential splice site nonsynonymous mutation, IVS5-2A>C segregates to the unaffected father while a heterozygous nonsynonymous nonsense mutation, c.1744G>T segregates to the unaffected mother and two unaffected siblings. Both mutations are present in all four affected foetuses. (B) Conservation of the mutated amino acid sequences are conserved amongst vertebrates.

Figure S4: Compound heterozygous mutations in *CENPF* cause primary microcephaly.

The heterozygous nonsynonymous nonsense mutation, c.1744G>T segregates to the unaffected father while the heterozygous nonsynonymous nonsense mutation, c.8692 C>T, p.R2898X segregates to the unaffected mother and two unaffected siblings but not the unaffected father. Both mutations are present in the patient exhibiting MCPH. Of note the unaffected father carries a known single nucleotide polymorphism c.8693G>A which is not present in the affected offspring with MCPH.

Figure S5: Western blot analysis of CENP-F protein derived from MCPH patient's fibroblasts revealed reduced protein levels compared to control.

SEE ALSO FIGURE S9B – HIGHLIGHTS RESOLUTION DIFFICULTIES IF DETECTING SMALL TRUNCATION OF PROTEIN OF 23kDa IF INCOMPLETE NONSENSE

MEDIATED DECAY AS A RESULT OF p.2898* MUTATION

Figure S9: Full length western blot analysis of CENP-F protein derived from MCPH patient's fibroblasts revealed reduced protein levels compared to control.

Figure S7

Quantitative graph demonstrating number of *cenpf* morphants exhibiting right-sided, left-sided, bilateral and absent southpaw mRNA expression compared to control embryos. Bars represent an average of three experiments. Error bars denote standard error of the mean (S.E.M). Std-MO (n=95 embryos) vs. cenpf-MO (n=101 embryos) mean left-sided expression 24±1.2 vs. 5±0.6, * $p<0.006$; mean right-sided expression 7±1.2 vs. 2±0.6, ** $p<0.01$; mean bilateral expression 10±1.2 vs. 2±0.6, *** $p<0.04$; mean absent expression 11±0.6 vs. 4±0.9, *** $p<0.04$

Figure S8:

Representative images of co-immunoprecipitation experiments carried out on protein lysates from unsynchronized RPE cells containing endogenous CENP-F. Immunoblots show that NuMA and Par 3 co-immunoprecipitates with endogenous CENP-F. IN= input; ten per cent of total input is indicated. (B) Representative images of co-immunoprecipitation experiments carried out on protein lysates from unsynchronized RPE cells containing endogenous p150 ^{Glued} subunit of dynactin and CENP-F. Immunoblots show that p150 ^{Glued} subunit of dynactin co- immunoprecipitates with endogenous CENP-F and CENP-F co- immunoprecipitates with endogenous p150 ^{Glued} subunit of dynactin. IN= input; ten per cent of total input is indicated.

Figure S9: Full length western blot analysis of CENP-F protein derived from MCPH patient's fibroblasts revealed reduced protein levels compared to control.
