# Supplementary material for: The kinetochore protein, CENPF, is mutated in human ciliopathy and microcephaly phenotypes
Source: J Med Genet. 2015 Jan 6;52(3):147–56. doi: 10.1136/jmedgenet-2014-102691 (PMC4345935; doi:10.1136/jmedgenet-2014-102691)
Supplement: Web figures [file jmedgenet-2014-102691-s2.pdf]

SUPPLEMENTARY FIGURE LEGENDS

Figure S1 (A) RT-PCR of RNA from cenpf splice zebrafish morphants demonstrating specificity of splice morpholinos by aberrant splicing of cenpf mRNA in cenpf splice morphants compared to control embryos at 24 hpf. (B) Zebrafish cenpf morphants exhibit high mortality in first 24 hours. Quantification (%) of surviving zebrafish embryos injected with morpholino (MO) at 24hpf. Graphic representation of results showing the mean percentage of survival at 24hpf of standard-MO (control), 1ng cenpf MO and 2ngs cenpf MO. Bars represent an average of three experiments. Error bars denote standard error of the mean (S.E.M). [Std-MO (1ng, n=266) % surviving embryos at 24hpf vs. cenpf-MO (1ng, n=173) 85.7±1.2 vs. 46.7±0.9, * p<0.008; Std-MO vs. cenpf-MO (2ng, n=256) 85.7±1.2 vs. 36.3±2.3, ** p<0.001; cenpf-MO (1ng, n=173) vs. cenpf- MO (2ngs, n=256) 46.7±0.9 vs. 36.3±2.3, ns, p<0.09].

Figure S2: Linkage plot of multipoint linkage analysis in a kindred with novel ciliopathy phenotype. Ten chromosomal regions were identified with a positive LOD score. Plot derived from GENEHUNTER version 2 [21].

Figure S3: Segregation and Conservation of compound heterozygous mutations in CENPF. (A) The heterozygous essential splice site

nonsynonymous mutation, IVS5-2A>C segregates to the unaffected father while a heterozygous nonsynonymous nonsense mutation, c.1744G>T segregates to the unaffected mother and two unaffected siblings. Both

mutations are present in all four affected foetuses. (B) Conservation of the mutated amino acid sequences are conserved amongst vertebrates.

Figure S4: Compound heterozygous mutations in CENPF cause primary microcephaly. The heterozygous nonsynonymous nonsense mutation, c.1744G>T segregates to the unaffected father while the heterozygous nonsynonymous nonsense mutation, c.8692C>T, p.R2898X segregates to the unaffected mother and two unaffected siblings but not the unaffected father. Both mutations are present in the patient exhibiting MCPH. Of note the unaffected father carries a known single nucleotide polymorphism c.8693G>A which is not present in the affected offspring with MCPH.

Figure S5: Western blot analysis of CENP-F protein derived from MCPH patient’s fibroblasts revealed reduced protein levels compared to control. SEE ALSO FIGURE S9B – HIGHLIGHTS RESOLUTION DIFFICULTIES IF DETECTING SMALL TRUNCATION OF PROTEIN OF 23kDa IF INCOMPLETE NONSENSEMEDIATED DECAY AS A RESULT OF p.2898* MUTATION

Figure S7: Quantitative graph demonstrating number of cenpf morphants exhibiting right-sided, left-sided, bilateral and absent southpaw mRNA expression compared to control embryos. Bars represent an average of three

experiments. Error bars denote standard error of the mean (S.E.M). Std-MO (n=95 embryos) vs. cenpf-MO (n=101 embryos) mean left-sided expression 24±1.2 vs. 5±0.6, * $p<0.006$; mean right-sided expression 7±1.2 vs. 2±0.6, ** $p<0.01$; mean bilateral expression 10±1.2 vs. 2±0.6, *** $p<0.04$; mean absent expression 11±0.6 vs. 4±0.9, *** $p<0.04$

Figure S8: Representative images of co-immunoprecipitation experiments carried out on protein lysates from unsynchronized RPE cells containing endogenous CENPF. Immunoblots show that NuMA and Par 3 coimmunoprecipitates with endogenous CENP-F. IN= input; ten per cent of total input is indicated. (B) Representative images of co-immunoprecipitation experiments carried out on protein lysates from unsynchronized RPE cells containing endogenous p150 Glued subunit of dynactin and CENP-F. Immunoblots show that p150 Glued subunit of dynactin co-immunoprecipitates with endogenous CENP-F and CENP-F co- immunoprecipitates with endogenous p150 Glued subunit of dynactin. IN= input; ten per cent of total input is indicated.

Figure S9: Full length western blot analysis of CENPF protein derived from MCPH patient's fibroblasts revealed reduced protein levels compared to control.

A.

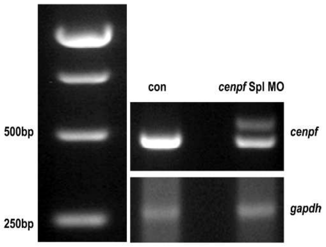

B.

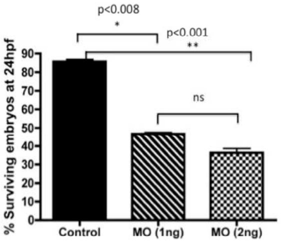

FIGURE S1

280x280mm (299 x 299 DPI)

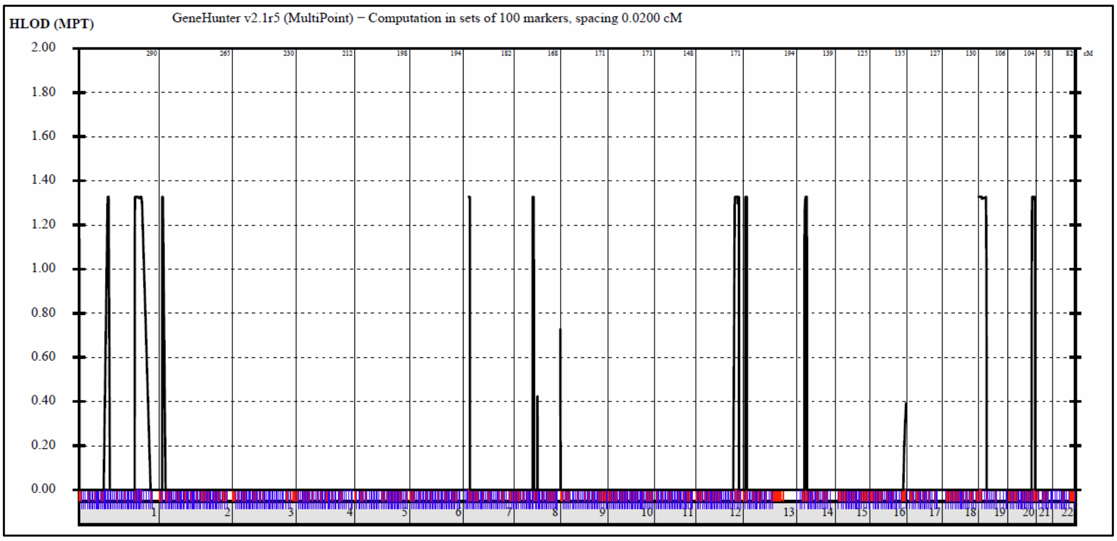
HLOD (MPT)
GeneHunter v2.1r5 (MultiPoint) − Computation in sets of 100 markers, spacing 0.0200 cM
2.00
1.80
1.60
1.40
1.20
1.00
0.80
0.60
0.40
0.20
0.00
290 265 230 212 198 194 182 168 171 171 148 171 194 139 125 135 127 130 106 104 58 82 cM
1 2 3 4 5 6 7 8 9 10 11 12 13 14 15 16 17 18 19 20 21 22

FIGURE S2

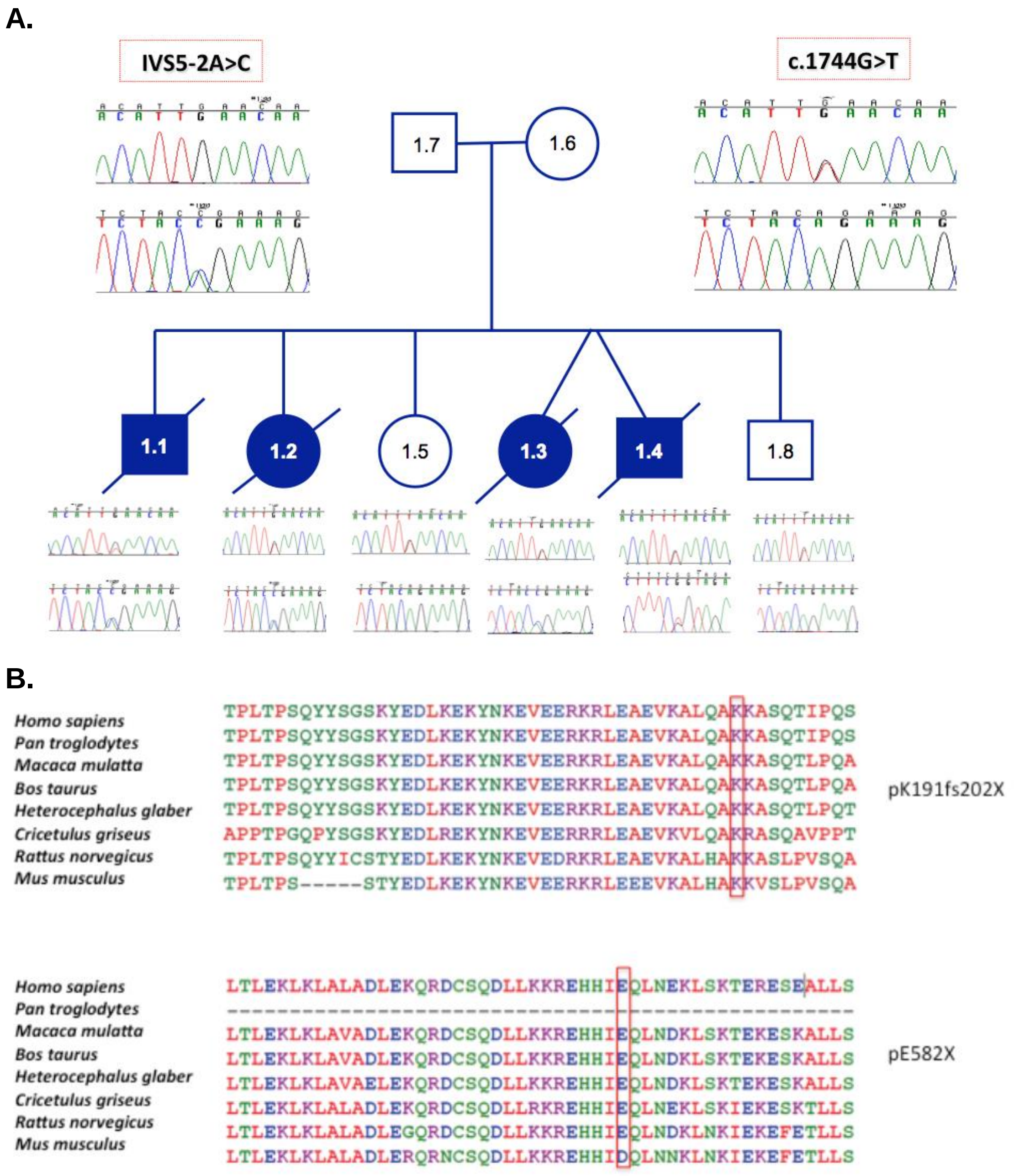
A.
IVS5-2A>C
c.1744G>T
1.7
1.6
1.1
1.2
1.5
1.3
1.4
1.8
B.
Homo sapiens TPLTPSQYYSGSKYEDLKEKYNKEVEERKRLEAEVKALQAKKASQTIPQS
Pan troglodytes TPLTPSQYYSGSKYEDLKEKYNKEVEERKRLEAEVKALQAKKASQTIPQS
Macaca mulatta TPLTPSQYYSGSKYEDLKEKYNKEVEERKRLEAEVKALQAKKASQTLPQA
Bos taurus TPLTPSQYYSGSKYEDLKEKYNKEVEERKRLEAEVKALQAKKASQTLPQA
Heterocephalus glaber TPLTPSQYYSGSKYEDLKEKYNKEVEERKRLEAEVKALQAKKASQTLPQT
Cricetulus griseus APPTPGQPYSGSKYEDLREKYNKEVEERRRLEAEVKVLQAKRASQAVPPT
Rattus norvegicus TPLTPSQYYICSTYEDLKEKYNKEVEDRKRLEAEVKALHAKKASLPVSQA
Mus musculus TPLTPS-----STYEDLKEKYNKEVEERKRLEEEVKALHAKKVSLPVSQA
pK191fs202X
Homo sapiens LTLEKLKLALADLEKQRDCSQDLLKKREHHIEQLNEKLSKTERESEALLS
Pan troglodytes --
Macaca mulatta LTLEKLKLAVADLEKQRDCSQDLLKKREHHIEQLNDKLSKTEKESKALLS
Bos taurus LTLEKLKLAVADLEKQRDCSQDLLKKREHHIEQLNDKLSKTEKESKALLS
Heterocephalus glaber LTLEKLKLAVAELEKQRDCSQDLLKKREHHIEQLNDKLSKTEKESKALLS
Cricetulus griseus LTLEKLKLALADLEKQRDCSQDLLRKREHHIEQLNEKLSKIEKESKTLLS
Rattus norvegicus LTLEKLKLALADLEGQRDCSQDLLKKREHHIEQLNDKLNKIEKEFETLLS
Mus musculus LTLEKLKLALADLERQRNCSQDLLKKREHHIDQLNNKLNKIEKEFETLLS
pE582X

FIGURE S3

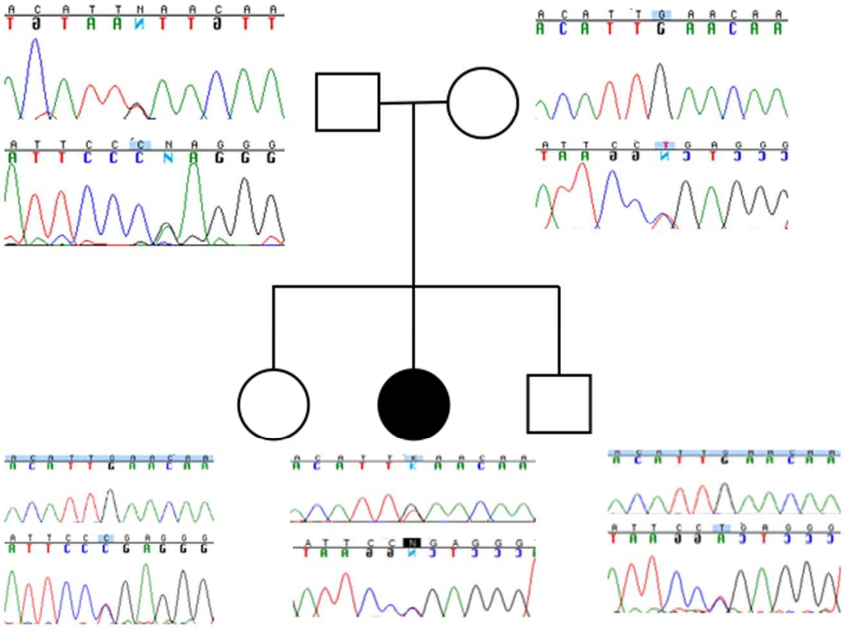

254x190mm (72 x 72 DPI)

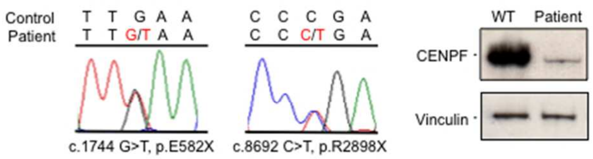

FIGURE S5

254x190mm (72 x 72 DPI)

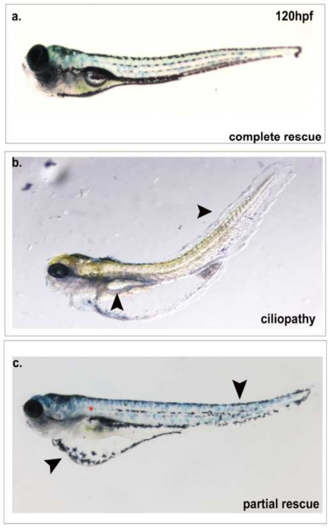
a.
120hpf
complete rescue
b.
ciliopathy
c.
partial rescue

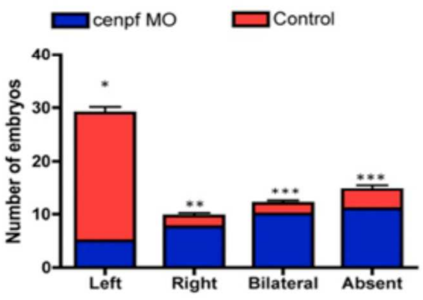
cenpf MO
Control
Number of embryos
40
30
20
10
0
*
**
***
***
Left
Right
Bilateral
Absent

Figure S7

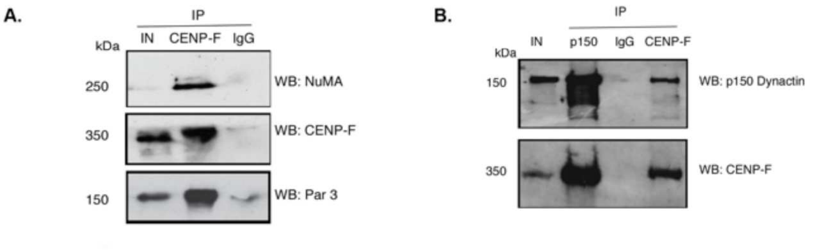
A.
IP
kDa
IN
CENP-F
IgG
250
350
150
WB: NuMA
WB: CENP-F
WB: Par 3
B.
IP
kDa
IN
p150
IgG
CENP-F
150
350
WB: p150 Dynactin
WB: CENP-F

Figure S8

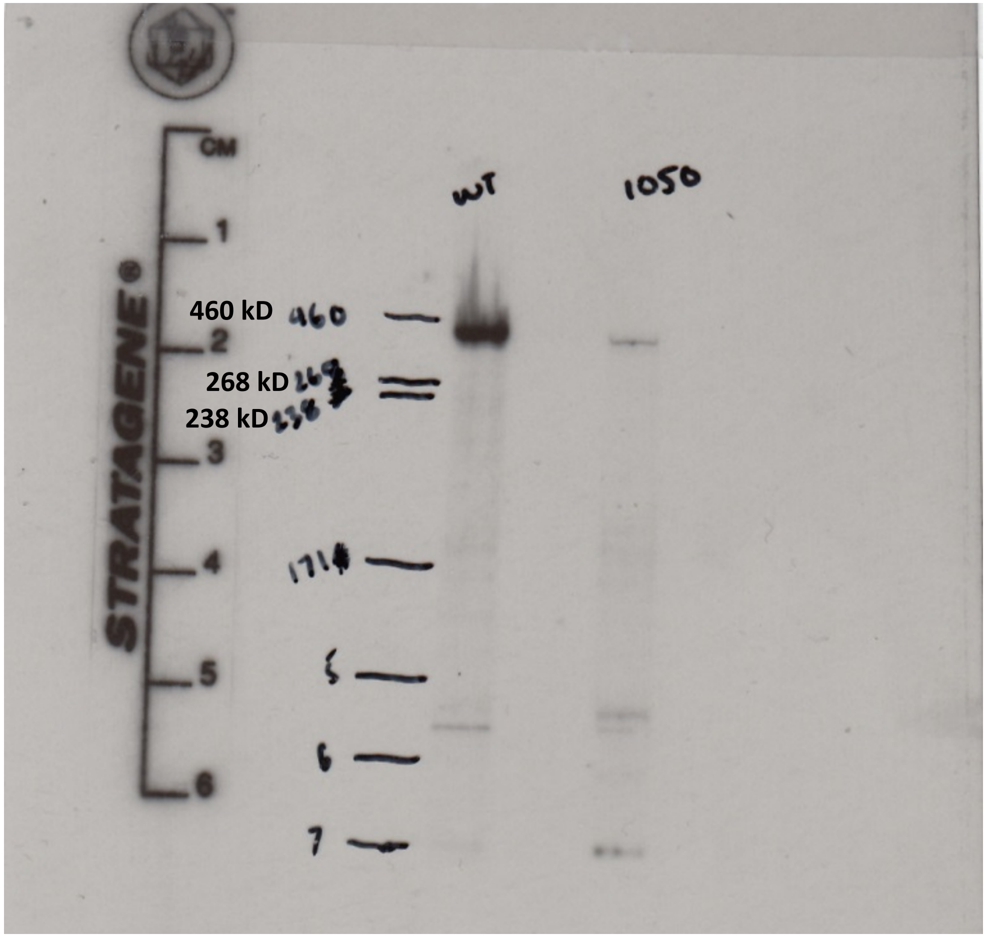

FIGURE S9
